# Using Persistent Homology as a New Approach for Super-Resolution Localization Microscopy Data Analysis and Classification of γH2AX Foci/Clusters

**DOI:** 10.3390/ijms19082263

**Published:** 2018-08-02

**Authors:** Andreas Hofmann, Matthias Krufczik, Dieter W. Heermann, Michael Hausmann

**Affiliations:** 1 Institute for Theoretical Physics, Heidelberg University, Philosophenweg 19, 69120 Heidelberg, Germany; Andreas.Hofmann@thphys.uni-heidelberg.de (A.H.); heermann@tphys.uni-heidelberg.de (D.W.H.); 2 Kirchhoff-Institute for Physics, Heidelberg University, Im Neuenheimer Feld 227, 69120 Heidelberg, Germany; matthias@krufczik.de

**Keywords:** single-molecule localization microscopy, DNA double strand breaks, γH2AX formation, persistent homology, topology, similarity measure, foci/cluster classification relative to heterochromatin

## Abstract

DNA double strand breaks (DSB) are the most severe damages in chromatin induced by ionizing radiation. In response to such environmentally determined stress situations, cells have developed repair mechanisms. Although many investigations have contributed to a detailed understanding of repair processes, e.g., homologous recombination repair or non-homologous end-joining, the question is not sufficiently answered, how a cell decides to apply a certain repair process at a certain damage site, since all different repair pathways could simultaneously occur in the same cell nucleus. One of the first processes after DSB induction is phosphorylation of the histone variant H2AX to γH2AX in the given surroundings of the damaged locus. Since the spatial organization of chromatin is not random, it may be conclusive that the spatial organization of γH2AX foci is also not random, and rather, contributes to accessibility of special repair proteins to the damaged site, and thus, to the following repair pathway at this given site. The aim of this article is to demonstrate a new approach to analyze repair foci by their topology in order to obtain a cell independent method of categorization. During the last decade, novel super-resolution fluorescence light microscopic techniques have enabled new insights into genome structure and spatial organization on the nano-scale in the order of 10 nm. One of these techniques is single molecule localization microscopy (SMLM) with which the spatial coordinates of single fluorescence molecules can precisely be determined and density and distance distributions can be calculated. This method is an appropriate tool to quantify complex changes of chromatin and to describe repair foci on the single molecule level. Based on the pointillist information obtained by SMLM from specifically labeled heterochromatin and γH2AX foci reflecting the chromatin morphology and repair foci topology, we have developed a new analytical methodology of foci or foci cluster characterization, respectively, by means of persistence homology. This method allows, for the first time, a cell independent comparison of two point distributions (here the point distributions of two γH2AX clusters) with each other of a selected ensample and to give a mathematical measure of their similarity. In order to demonstrate the feasibility of this approach, cells were irradiated by low LET (linear energy transfer) radiation with different doses and the heterochromatin and γH2AX foci were fluorescently labeled by antibodies for SMLM. By means of our new analysis method, we were able to show that the topology of clusters of γH2AX foci can be categorized depending on the distance to heterochromatin. This method opens up new possibilities to categorize spatial organization of point patterns by parameterization of topological similarity.

## 1. Introduction

DNA double-strand breaks (DSBs) can be induced by ionizing radiation and are known to be the most severe damages in the genome of a cell nucleus. The amount of DSBs simultaneously occurring is dependent on the radiation dose, the LET (Linear Energy Transfer) of radiation, the cell type, the radio-sensitivity of the cell, etc. Recent investigations have shown that DNA damaging is accompanied by an instant spatial reorganization of chromatin at and around the damaged site [1,2,3] and an activation of the repair machinery. One of the first steps of chromatin modification after DSB induction is phosphorylation of the histone variant H2AX [4] to γH2AX within a given neighborhood of the damaged site [5,6,7]. Such foci seem to “tag” the locations of damaged DNA for the recruitment of proteins that are starting and processing the follow-up repair [8,9]. At that point, a decision about the next procedure has to be made by the cell [10]. Several factors such as cell cycle state, functional activity of genes, break position along the DNA sequence, temporal state of DNA compaction, number of simultaneously occurring DSBs, etc., are known to influence this decision and the consequences for a cell nucleus and the genome [10,11,12].

At a very first glimpse, the cell has to decide between fast or error-free repair for each DSB within the first minutes after damaging by irradiation. One choice may be homologous recombination repair (HRR) [11], which is a rather slow but error-free repair process. HRR needs an intact DNA sequence template of the homologous chromosome, along which a complementary strand can be reconstructed. In contrast to HRR, non-homologous end joining (NHEJ), a very frequently used repair process, may cause errors in the DNA base sequence but works much faster than HRR. Several specific proteins process the broken DNA ends by strand resection and re-connection of the broken ends at appropriately complimentary bases. HRR and NHEJ are the most often chosen pathways (for review, see [12,13]). HRR may sometimes be suppressed within repetitive DNA units if the damaged DNA side is not relocated to the heterochromatin periphery. In these cases, single-strand annealing (SSA) takes place instead [14]. It has also been shown that especially in cases of irradiation at higher doses (>2 Gy) and consequently more DBSs, the conventional NHEJ (c-NHEJ) may fail at some breakage sites and an alternative NHEJ process (a-NHEJ) is applied, which is a slow and error-prone repair process [13,15].

On the one hand, HRR may be the first choice and preferentially used to keep the genome as preserved as possible. Only if HRR is insufficient (e.g., due to too many DSBs at higher doses) then NHEJ saves the situation since it is much faster. Recently, it has been shown that in G1 resection dependent NHEJ is possible, which seems to be different from resection processes in HRR [16]. On the other hand in G2, most DSBs are repaired by NHEJ. So some people believe that NHEJ is always the first choice and only in those cases where NHEJ fails, HRR saves the repair [17].

Each of these different repair processes requires a different cascade of proteins that are time dependent recruited and decruited during the repair process and are responsible for DNA strand end clipping and processing, end-to-end fixation, or correct sequence re-association [18,19]. Although many steps of DNA strand processing and its relevant proteins are known and the interaction of proteins during the different pathways are often well understood, the question as to what makes up the cell’s decision for a certain pathway at a certain damage site remains insufficiently answered. Considering all the major factors that influence the repair pathway choice and the quickness of the cell response, this may suggest a still unknown or not sufficiently understood central mechanism behind the pathway choice. This mechanism should work at each damaged side individually. This means that physical as well as topological parameters of the DNA strand break environment may determine the repair pathway choice together with epigenetic conditions [8,9]. This assumption has been recently supported by investigations showing that radio-sensitivity can be modulated by chromatin remodeling in daughter cells of irradiated samples [20].

Assuming that the genome architecture and the architecture of repair complexes on the micro-and especially on the nano-scale become important for a repair focus region, not only novel techniques for a detailed analysis of spatial foci organization are required but also methods to categorize foci or sub-foci (clusters) and to compare each focus/cluster with each other independently of the cell or cell nucleus. Nano-scaled analysis has reasoned several transmission electron and super-resolution light microscopic studies in order to elucidate the spatio-temporal internal organization of repair foci and their chromatin surroundings with molecular resolution [3,21,22,23,24,25,26,27,28,29]. Recently, it has been shown by super-resolution light microscopy, that γH2AX foci are built up by clusters that form nano-foci with different repair activities [23,28] and that inside these nano-foci repair proteins are well organized [23,24,29] whereas the chromatin environment is interacting in a characteristic arrangement [28,30,31]. In addition, it has been shown that after radiation exposure and DNA damaging, Alu heteroduplexes may undergo Alu/Alu recombination into a single chimeric Alu element by NHEJ [32]. This may reason a dose dependent accessibility of ALU-sequence specific oligonucleotides (17mer uniquely binding to the ALU consensus sequence) as detected by SMLM [33,34].

During recent years, it has been demonstrated that single molecule localization microscopy (SMLM) [35] is an appropriate technique to elucidate conformations of molecular arrangements and their functional relevance in cell nuclei, cytosol, and on cell membranes [1,28,29,30,31,33,34,36,37,38]. An embodiment of SMLM [39] as being used in this article, applies standard fluorescent dyes for specific labeling that can be switched between spectral “on” and “off” states [40,41] to spatial separation of molecules (“reversible photo-bleaching”). From a reversible dark state, the fluorescent molecules randomly return to the emission state and cause blinking events that can be separated from a continuously fluorescent background. Each position of an emitting fluorophore is represented by an Airy disc and can precisely be located as the center-of-mass (barycenter) of such a disc. This also allows the precise calculation of spatial distances between fluorescent molecules in the 10 nm regime [34,42,43]. Using the matrix of the coordinates of fluorescent tags, all acquired positions can be visualized by an artificial “pointillist”, super-resolution image. In the images representing the point distribution, the effective resolution is only depending on the localization precision [43]. Moreover, the images can also encode results of distance analysis evaluations or density measurements.

However, localization data sets (e.g., labeling molecules of γH2AX or methylation sides of heterochromatin such as H3K9me3) consist of tens or even some tens of thousands of individual point coordinates and their visualization and analysis is a separate challenge, since a point pattern does not automatically reveal a characteristic conformation or shape. In that way SMLM data fundamentally differ from conventional microscope images. While a conventional microscope provides an image with contours resolved with a scale of the order of 100 nm, SMLM is only producing a coordinate matrix with the positions of the fluorophores in the nanometer range. Such a pointillist representation requires a new approach to extract the relevant conformational information, in such a way that the point distribution is unequivocally transferred into a certain shape or better topology that may be also maintained under different perspectives and different deformations. This requires quantitative analysis using mathematical concepts.

Approaches for a quantitative point density, distance, or cluster analysis exist for SMLM [23,28,29,33,34]. The analysis is restricted in scale to a certain order of magnitude and does not consider shape deformation. Quantifications on several orders of magnitude (for example in the range of a few nanometers up to several hundred nanometers) are hardly possible and cannot be easily compared according to typical characteristics. In order to overcome these restrictions, a novel mathematical approach is presented here, which analyzes SMLM data with methods of persistent homology [44]. This has the advantage that both the geometric and the topological properties of given point distributions are considered [45] and a parameter-free quantification of the structural arrangement of a point distribution over several orders of magnitude is possible. Thus, the accuracy achieved by state-of-the-art SMLM can be used, not only for a point pattern analysis, but also for a structural analysis of molecular arrangements. The point distributions and thus the underlying structures (e.g., heterochromatin distributions or γH2AX foci/clusters) can now be directly compared independently of a cell nucleus whereby both nano-scale and micro-scale level differences are considered. Mors theory and set theory allow for a quantitative comparison of two point distributions and a categorization according to a similarity measure. This higher degree of abstraction compared to image visualization achieves a higher degree of information and functionally relevant insights. 

In order to demonstrate the power of this new mathematical approach for SMLM data, a proof of principle has been applied to analyze and categorize clusters of γH2AX repair foci according to their structure and chromatin vicinity. The packaging degree of the DNA has consequences for the repair process. This is especially true for the densely packed heterochromatin because the damaged DNA has to be histone free for the repair and must also be accessible for the repair protein complexes [46,47,48]. It has been shown that DSBs in the heterochromatin region are usually be repaired at the border of heterochromatic chromatin regions [1,2,14,49] whereby the methylation degree typical for heterochromatin remains unchanged. Re-organization within heterochromatic regions is necessary to make the damage accessible for repair proteins. Therefore the proximity to heterochromatin was the parameter that was correlated to the internal topology by means of the topological data analysis (TDA). The topological representation of each focus was compared to each other and the degree of similarity was determined.

## 2. Results

In the following section, the mathematical approach is described with the aim to show the requirements for the γH2AX foci/cluster analysis.

### 2.1. SMLM Data Processing

The raw data obtained by SMLM consist of a stack of about 2000 image frames acquired in a continuous time series. The blinking events registered above the fluorescent background are used to compute the exact positions of the individual fluorophores. Tests during former experiments [28,29,34] have revealed that the intensity of the maximum of a point signal must be at least four times higher than the background intensity to get registered as an event. The double of the average background intensity is subtracted from each pixel. The intensity barycenter µ and the associated standard deviation σ of each signal are determined using a two-dimensional Gaussian function f. Finally, the localization precision ∆µ can be calculated that depends, among others, on the specificity and accuracy of labeling, on the number of detected photons q_i_ and the background intensity N_B_. A more detailed description can be found in [33,34,38]. The results of the data acquisition are matrices containing the coordinates for each measured fluorescent point and the localization precision of each point. From such a matrix, data can be evaluated and structures can be interpreted as well as a pointillist image can be produced. As being an artificial image, results of data processing can also be used to code the image points. 

**Figure 1:**
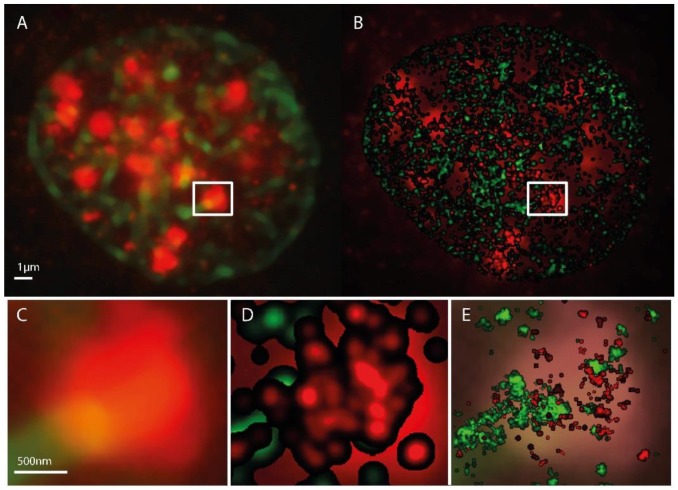
Microscopy images of H3K9me3 (green) and γH2AX (red) immunostaining in a SkBr3 cell nucleus 30 min after irradiation with 1 Gy, 6 MeV photons. (**A**) conventional widefield microscopy image. (**B**) SMLM image represented as a density image where the brightness of a point refers to the number of next neighbor points. In the background the conventional widefield microscopy image of γH2AX fluorescence is shown. The SMLM image is created from the coordinate matrix where the pixel geometry and intensity is stored in pixel values. After determination of the pixel size in nm, the coordinates and distances can be evaluated. In a fixed radius R around each coordinate, it is determined how many further coordinates are within this radius. This value is coded in the intensity of the coordinate point. In order to emphasize contiguous structures, each coordinate with an assigned value greater than zero is the starting point of a Gaussian distribution with a given sigma. The sum of all Gaussian distributions then represents the intensity distribution of the Gaussian-filtered density image (example: pixel size = 10 nm/pixel, Radius R = 1000 nm, Gaussian filter σ = 50 nm). (**C**) magnified insert from (**A**). (**D**) magnified insert of (**B**). (**C**,**E**) superposed by the standard localization image. This supposing image is directly created from the localization data. In-homogeneities and sub-structures within the γH2AX cluster are visible. Again a pixel size has to be determined. Here, every coordinate is starting point of a Gaussian distribution with the localization precision as sigma (pixel size = 10 nm/pixel).

In Figure 1, representative images are shown comparing localization microscopy results with microscopic visualization. Here, an example of an irradiated cell is shown. For comparison, an example of an untreated cell is shown in the supplement (Figure S1) indicating that only background or very few γH2AX clusters of the same size as in irradiated samples are visible (Figure S2). It should be mentioned that for normal light microscopy, the image is reflecting the visual impression whereby for SMLM, the image is the result of data processing. This means that in SMLM data can be analyzed without an image and thus data analysis is independent of any image and from procedures of computer image analysis.

### 2.2. γH2AX Cluster Recognition and Cluster Classification

SKBr3 is known being a cell line of increased radio-resistance with well separated γH2AX foci also after radiation treatment with doses of several Gy. It has been shown that such foci can be separated into functionally relevant sub-foci or clusters of about the same size independent from the dose applied [23,28,29]. This has also been found for the cell nuclei analyzed here (see Figure S2). Depending on the dose applied, the clusters are relaxing during the repair time (for details see [28]). Therefore cluster formation in γH2AX foci was determined at 30 min post irradiation, i.e., at an early repair time, but for different doses (see Materials and Methods). 

A cluster analysis software was applied to the localization data of γH2AX foci. The algorithm identifies points referring to a cluster within all the localization data according to user-defined parameters that were iteratively determined. These parameters are the minimum number of neighboring fluorescence signals within a defined radius around each labeling point and this given radius. A labeling point is identified as a member of a cluster if at least a minimum number of points are located within the predefined radius. The remaining points are identified as outside cluster points (“noise-points”). If two cluster points have a smaller distance than the given radius, they belong to the same cluster. All points whose distance to a cluster point is smaller than the radius also belong to the cluster. This cluster search algorithm is called “Density-Based Spatial Clustering of Applications with Noise” (DBSCAN) [50]. In this case, this allows the identification of γH2AX-dense regions. Here, a radius of 200 nm and a minimum point number of 50 was used to identify “γH2AX clusters”. These clusters are not identical with γH2AX foci obtained by diffraction limited wide-field imaging. It has been shown that the foci are sub-divided into several sub-units which are compatible to the clusters described here [23,28]. Furthermore, the two parameters for the cluster recognition have been varied in order to ensure that the results of the subsequent analysis discussed in the following are not crucially dependent on this choice of parameters.

**Figure 2:**
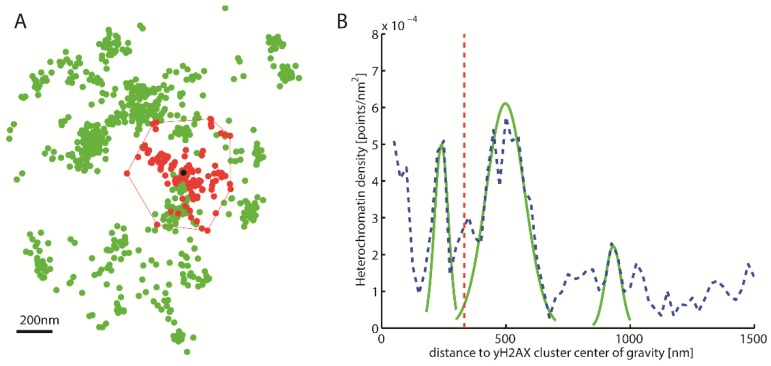
Density distribution around γH2AX cluster. (**A**) Schematic representation of a γH2AX cluster (red) recognized by Density-Based Spatial Clustering of Applications with Noise (DBSCAN), the center of gravity (black) and the heterochromatin (green) around it. (**B**) Density distribution of heterochromatin around the γH2AX cluster center of gravity (blue). This density distribution is used to classify whether a cluster is heterochromatin-associated. For this purpose, the local maxima are approximated by Gaussian functions (green). The amplitude of these maxima is then compared with the predetermined threshold value. The red line represents half the mean square distance of the convex hull of the cluster (i.e., a kind of “radius” of the cluster).

After cluster recognition, the centers of the γH2AX clusters were computed with the Surveyors Area Formula [51]. These centers were used as the center points of increasing circular shells. The heterochromatin density in these shells was computed (Figure 2) (heterochromatin density equals the number of heterochromatin points per area of the corresponding shell).

Based on this heterochromatin density distribution tagged by antibodies against H3K9me3, the γH2AX clusters obtained from several cell nuclei exposed to different radiation doses were divided into heterochromatin-associated ones (HC) and non-heterochromatin-associated ones (nHC) clusters. For this purpose, the local maxima of the density distribution were determined (see also Figure 2B); thereby the expansion of the circular shells necessary for determination of the density distribution affects this classification. A step size being too small (e.g., 1 nm) leads to a distribution with very many local maxima and minima since the density can change significantly in such small circular shells. Due to the small extent, it is possible that there is no point in a circular shell and a certain number of points in the nearest one. Due to the small surface area of the circular shells, considerable jumps are produced, which are methodically caused and only represent the real density distribution poorly. However, a step size being too large results in less separable characteristic peaks of the mapped distribution. After some iteration, a step size of 25 nm was chosen for density analysis. This step site ensures that the major local maxima are recognizable within a density distribution of heterochromatin points without losing the characteristics of the distribution (see also Figure 2B). 

For the applications shown here on SkBr3 cells, a minimum for the amplitude of a local maximum of at least A_min_ = 2.5 × 10^−4^ points/nm^2^ was determined in pre-experiments of automatic cluster search. The local maxima should have been spaced by at least 150 nm from each other in order to avoid overlapping maxima. Clusters with a local heterochromatin density equal or higher than A_min_ within a radius R = 250 nm were assigned as HC clusters. If the distance of the density maximum to the center of the cluster was larger than 250 nm, the clusters were assigned as nHC clusters. R = 250 nm is a suitable discrimination threshold when analyzing heterochromatin densities at the border of γH2AX clusters in HeLa cells (for details see [28]). It must be noted that the central range of a γH2AX cluster, i.e., a radius from 0 to 50 nm is excluded from the determination of the heterochromatin density distribution in order not to over-estimate no or a few labeling points on a very small central area. Such densities can only lead to extreme values, i.e., either zero (if a cluster does not contain any heterochromatin labeling point) or a large number (if some points are on a very small area in the cluster). Furthermore, it is assumed that in the center of a cluster the major repair activity takes place so that these labeling points may be due to relaxed and highly compacted heterochromatin. 

### 2.3. Topological Analysis of the Clusters

SMLM data of H2AX phosphorylation were analyzed by means of persistent homology, a method for computing topological features at different spatial resolutions. In particular, the structure of each γH2AX cluster was characterized by its so called α-shape. In the following, we introduce the computational strategy.

#### 2.3.1. Barcodes as a Representation for a Pointillist Structure

A major principle to characterize the meaning of “topology” or “topological analysis” is to record properties of structures (depicted in a pointillist manner) which are invariant under certain deformations of the object. Mathematically these deformations correspond to continuous transformations of the topological space defined by the structures. Deformations which might fragment the structures are excluded. In the following, the attention will be focused on two quantifiable properties: (a) the number of components which are independent from each other in such sense that connections between points only exist within the respective components; (b) the number of holes of the structures inside the components. In algebraic topology, these properties are called the Betti numbers for zero dimensional and one dimensional simplicial complexes, respectively. They turn out to be very important topological invariants which help to distinguish between different topological spaces.

By comparing these quantities to two objects, it can be decided whether they have the same topology or not. Localization microscopy images are actually point-sets defined by the location of the fluorophores. Thus an appropriate method is required by which components and holes can be defined. In order to accomplish this, the point-set is converted into an object as described by the following procedure: First, the point-set is defined by the coordinates of blinking labeling points. In the next step, a geometric relationship among the points is defined by growing spheres of radius α around each of them. Whenever two spheres mutually embed each-other’s center, these centers of the growing spheres are connected by an edge. Points connected in that way are considered to belong to the same component. Any two points which are connected by a path through the existing edges are in the same component. Increasing the radii of the spheres, further points are reached connecting two previously disjoint components. Thus one can follow how the number of components is changing as a function of the increasing radius α. This means that each point is a separate component at the beginning, whereas for an increasing radius being large enough, each point is connected with each other. At the end of the procedure a single component is remaining. 

**Figure 3:**
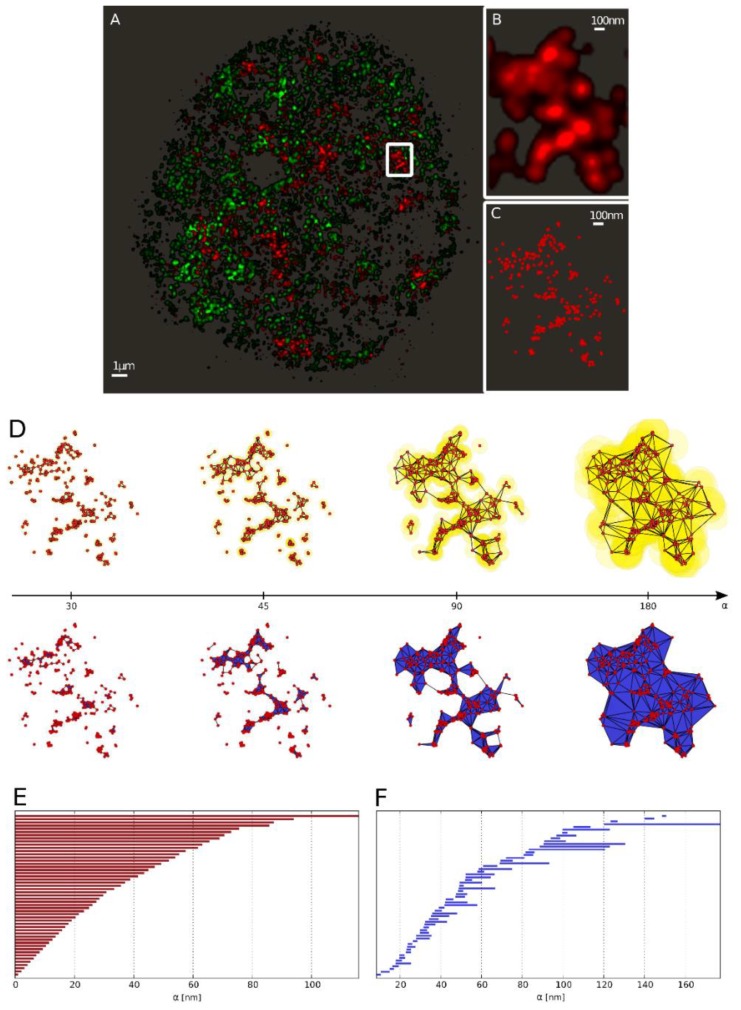
From the single molecule localization microscopy (SMLM) data to the barcode representation. (**A**) Full view of heterochromatin (green; labeled by antibodies against H3K9me3) and γH2AX labeling (red) of a SkBr3 cell nucleus 30 min after irradiation with 2 Gy depicted as a density image. (**B**) Zoom-in into the marked γH2AX cluster. (**C**) Scatter plot of the marked γH2AX cluster (every point represents a detected fluorophore). (**D**) Components of the α-shape filtration of the marked γH2AX cluster exemplarily depicted at α = {30, 45, 90, 180} nm (left to right). As the growing spheres mutually embed the center of each-other the corresponding centers are connected by an edge (as shown in the upper row). Whenever a triangle is formed, it is included in the solid as a face element (illustrated in the lower row). (**E**) Barcodes of dimension 0 (Betti number) corresponding to connected components. (**F**) Barcodes of dimension 1 (Betti number) corresponding to holes.

The definition of holes also stems from this process. In order to build a solid, beside points and lines, face building blocks are required. For this, the simplest polygon, the triangle is appropriate. Whenever three edges form a triangle, not only the edges but the face of the triangle is considered. The described procedure is presented in Figure 3D for a particular γH2AX cluster. Once the surfaces are defined, the holes are counted. In fact, it is possible to register their number and the number of components for every separate value of the radius α.

In [44], an approach is presented that shows how all components and holes can be summarized in a compact way. The presented approach allows for the representation as “barcodes” to track the formation and disappearance of components and holes as the value of α increases and thus independently of a fixed value of α. An example is shown in Figure 3E,F. The beginning of a line in the barcode representation shows at which value of the radius α, the component or hole has arisen, and the end of the line for which value of α it has disappeared as a result of association to a larger component. All red bars start at zero, because at a value of α = 0, all points are unconnected and for that reason each represents its own component. Whenever two points are joint to a line (or three points to a triangle), the two (or three) points are combined in the newly created component “line” (or “triangle”), and hence, the associated red bar ends. By further joining points with increasing value of α, the lines, triangles and holes shown in Figure 3D are created. The lifetime of a hole is represented in Figure 3F by blue bars. A bar starts when the hole is created and ends when it is completely filled.

The barcode thus represents an image of the examined structure on all scales. The creation and dissolution of complexes on a small scale is recorded alongside the lifetime of complexes on larger scales. As a consequence, in the case of chromatin structure or γH2AX cluster structures, barcodes contain information about components and holes in both the nanometer and micrometer scale ranges. This compact and illustrative representation also allows selection of specific substructures, such as all components and holes that exist in the range of α_1_ to α_2_.

As illustrated, the characterization of point structures by barcodes opens up new possibilities to analyze and categorize clustered structures in cell nuclei. Although, the barcode representation of a point-set might appear at least at first glance as confusing as the point-set itself, the possibility to compare different sets of barcodes and to define parameters describing their similarity is a significant advance in the analysis of point structures.

#### 2.3.2. Similarity of Barcodes

In study [45], an approach is presented that can be used as a measure of the similarity *S* of barcodes. The similarity of two barcodes *A* and *B* of given dimension, which are comprised of bars *a* and *b*, respectively, is then represented by:

$$S\left(A,B\right)=\frac{1}{\left|A\right|+\left|B\right|}\left[\underset{a\in A}{\sum }\underset{C}{\underbrace{sup_{b\in B}\frac{\left|a\cap b\right|}{\left|a\cup b\right|}}}+\underset{b\in B}{\sum }\underset{D}{\underbrace{sup_{a\in A}\frac{\left|a\cap b\right|}{\left|a\cup b\right|}}}\right]$$

*J*(*a*,*b*) *= |a∩b|*/*|a∪b|* represents the Jaccard index [52], which is a measure of the similarity of two bars. The result is a value between 0 and 1, where a value of 0 means no overlap of the two bars and two identical bars have a value of 1. The similarity measure for barcodes is described by the formula *S*(*A*,*B*). The part marked C in equation *S*(*A*,*B*) states that for every bar *a* the bar *b* is searched, for which the Jaccard index *J*(*a*,*b*) is maximized. This is repeated for each *a* and summed up. Analogously, for each bar *b* the bar *a* is searched, for which the Jaccard index *J*(*a*,*b*) *is* maximized. Again, the results for the individual bars *b* are summed up.

**Figure 4:**
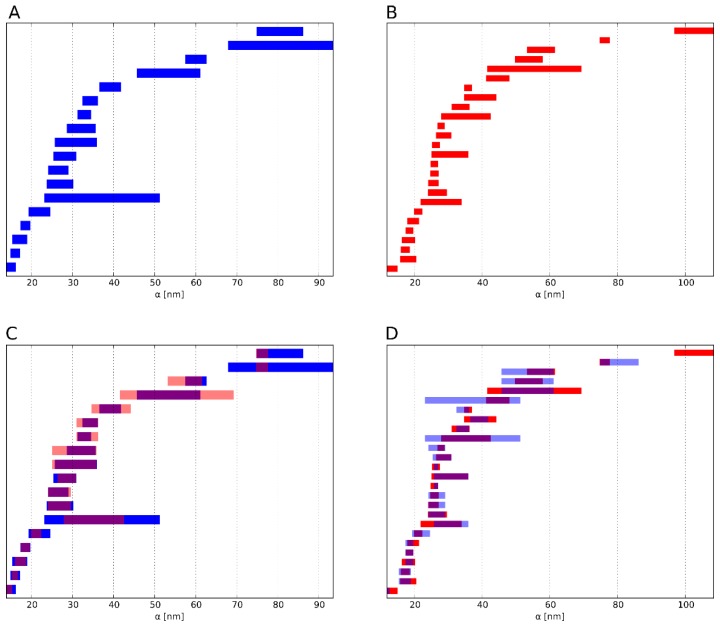
Example of the result of the barcode similarity measure. For each bar of barcode (**A**) (blue), the bar from (**B**) (red) is sought for which the Jaccard index is maximal. (**C**) The Jaccard index represents the extent to which two bars overlap (pink). This corresponds to C in equation *S*(*A*,*B*). These values are summed for each bar of A. (**D**) Analogously, for each bar from B the bar from A is sought, for which the Jaccard index becomes maximal. This corresponds to D in equation *S*(*B*,*A*). These values are in turn summed for the bar of B. The sum of these two subtotals is divided by the number of bars in both barcodes. The result *S*(*A*,*B*) or *S*(*B*,*A*) quantifies the similarity of the barcodes A and B in terms their overlap. Here, two barcodes of dimension 1 (holes) with a high similarity are depicted, i.e., the overlap of the two sets of barcodes is high. For comparison, we illustrate two dissimilar barcodes of dimension 1 in the supplementary material (see Figure S3).

The resulting sums are then added up and divided by the total number of bars of the two barcodes. As a result of the division, the similarity measure *S*(*A*,*B*) can vary between 0 and 1. An illustrative description based on two example barcodes is shown in Figure 4.

The similarity of barcodes of different dimensions can be defined as the average of the individual similarity values.

### 2.4. Proof-of-Concept Experiments

From the γH2AX clusters classified, 200 HC clusters and 200 nHC clusters were selected by determining those with the highest and lowest heterochromatic densities, respectively. This number of clusters was chosen because this group is large enough to avoid statistical outliers, but small enough to visually check microscopy images as obtained for each cluster from the point matrices and, if necessary, the corresponding density distribution.

The HC- and nHC-associated γH2AX clusters were examined according to their topological similarity as defined by the overlap measure described above. For comparison, the similarity between the two groups of γH2AX clusters was also determined in terms of density and size. Since the clusters are polygonal, the root mean square of the distances of the points of the convex hull has been defined as a measure of cluster size.

**Figure 5:**
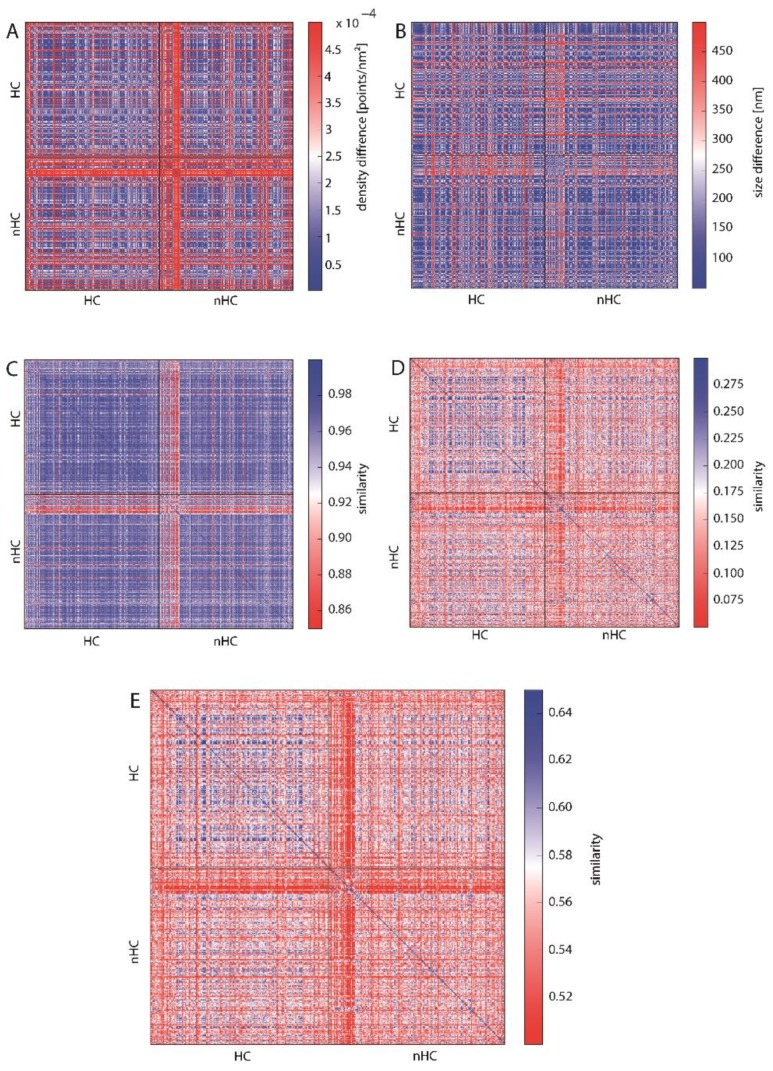
Heat maps depicting the similarity of HC- and nHC-associated γH2AX clusters according to (**A**) cluster density, (**B**) cluster size indicating a high similarity (see also Figure S2), (**C**) topological similarity of the connected components (“lines”), (**D**) topological similarity of the “holes”, (**E**) average topological similarity. The spectrum of the color bar of all the heat maps ranges from red representing dissimilarity between the analyzed clusters to blue depicting similarity.

To enable a comparison of all γH2AX clusters, the values of the topological similarity measure are depicted in a heat map (Figure 5). The arrangement of the heat maps is as follows: The upper left quarter compares the HC clusters with each other, the lower right the nHC clusters. The upper right and lower left quarters each compare nHC with HC clusters. The arrangement of the individual HC and nHC clusters is random. The spectrum of the color bar of all the heat maps ranges from red representing dissimilarity between the analyzed clusters to blue depicting similarity. In this representation, it is then easy to detect certain patterns, such as similarity of all the foci clusters, outliers or distinct areas of increased similarity or rather dissimilarity. As a measure of the similarity or rather dissimilarity of density (or size), the difference in density (or size) of two clusters was used. A small difference here means a great similarity.

In Figure 5A the difference in point densities between the analyzed clusters is presented. There is no clear difference between the clusters assigned to HC or nHC with most of the clusters showing similar density. However, there is a set of nHC clusters which are highly dissimilar to all the other clusters. This is shown by the red cross in the middle of the heat map and can be explained by the fact that these nHC clusters are significantly smaller than the median cluster size (see Figure 5B and Figure S2). Small clusters have a higher density than the other cluster. Analogously, Figure 5B does not reveal a difference between HC and nHC cluster. Hence, in terms of density and size of the foci, the proximity to heterochromatin does not appear to affect differences for the clusters.

Figure 5C–E show the similarity of the clusters using the topological similarity measure presented above. Here, cluster structures are characterized by barcodes for connected components and holes (Figure 3 shows the actual barcodes for one γH2AX-cluster). Surprisingly, the clusters show nearly identical results for the components of interconnected points (Figure 5C). Once again, the crosses are visible, which refer to extremely small clusters as it has already been explained above in the heat maps depicting the density. These aberrant clusters may be sorted out and further analyzed by repair protein staining. Maybe these clusters refer to such components that do not contain any repair activity, as it has recently been shown by Natale et al. [23]. But this should be tasks for further investigations.

Comparing the barcodes of dimension 1 representing holes (Figure 5D), the HC assigned clusters show a higher similarity in contrast to the nHC clusters, although the topological similarity values are very low. This, however, can be adjusted by using the average similarity for components and holes (Figure 5E). After this averaging, HC assigned clusters show a clear similarity whereas the nHC assigned clusters do not. This means that by topological analysis the HC clusters may be discriminated as those clusters of high topological similarity. The proximity of γH2AX clusters to heterochromatin seems to have a significant and measurable impact on its structure. Interestingly, the nHC and HC clusters are more similar than the nHC foci themselves. It can be clearly seen that, on the one hand, the proximity to heterochromatin influences the structure of the foci, but on the other hand that there are other γH2AX cluster influencing factors, otherwise the similarity between nHC and HC foci would be unexplainable.

## 3. Discussion

DNA double strand repair uses fascinating mechanisms that have been developed during evolution towards two different directions fast and error tolerable or slow and exact [12,13,14,15]. After having induced a DSB by ionizing radiation, chromatin re-arranges and H2AX phosphorylation occurs in the damage environment [1,2,28] within a few minutes accompanied by the recruitment of proteins specific for a certain repair mechanism. During the last decades modern techniques applied in radiation biology and radiation biophysics, have offered detailed insights into the protein interactions and cascades along the different repair pathways [8,9]. These investigations have completed our understanding about repair processes and boundary conditions that favor repair towards either end-joining processes such as NHEJ or recombination processes such as HRR. The better our understanding has become the more the question becomes urgent how a cell can decide which repair pathway should be the appropriate one at a certain damage side. Cells can simultaneously use all repair pathways in a cell nucleus at different damaged sites.

The repair pathway choice could be random for instance. This, however, is not convincing since it has been shown that whenever it is functionally relevant for cell survival a fast repair process is addressed.

Assuming a non-random pathway choice at a given damaged side raises the question for a fast, easy and therefore always functionally available, and everywhere implemented mechanism for the cell’s decision. Beyond several epigenetic approaches, people have started to discuss whether such a mechanism may be encoded in the architecture of chromatin around the damaged site (key note) lectures and discussions at the joint ERRS (European Radiation Research Society) and GBS (Gesellschaft für Biologische Strahlenforschung) conference 2017 in Essen, Germany). This would, however, require deeper insights into the internal structural organization of a repair focus of a typical order of size of about the resolution limit of a light microscope (about 200 nm).

Recent applications of electron-microscopy [25,26] and super-resolution light microscopy such as SMLM, STED or GSDIM [1,3,23,24,28,29,30,31,33,34] have demonstrated that it is feasible to study single molecular arrangements within a repair focus. With improving resolution of microscopy and data evaluation of structures on the meso- and nano-scale, the question for best suited analysis parameters and potentially useful classification criteria of repair foci and damaged chromatin sites has become important. 

Here, we have introduced a rather unconventional approach for SMLM data analysis of γH2AX foci and their chromatin environment. This approach makes use of the advantage that SMLM data can be evaluated without image production and image processing [34]. This novel approach combines a geometrical evaluation based on Ripley’s distance and cluster analysis with persistence homology for similarity classification of repair cluster loci. Although the mathematical principles behind this approach are well established, it is the first time that topology has been used as biologically relevant criteria. This may allow to circumvent locally occurring deformations in the analysis and to extract a parameter pattern that is scale independent and can categorize repair foci into structural classes. Here we have demonstrated a very first proof-of-concept experiment, in which we could show that the category of HC associated γH2AX clusters are highly similar in terms of both topology and geometry whereas nHC associated clusters are completely dissimilar. This topological similarity was independent of the irradiation doses. However, only one early repair time was considered. In future experiments other later repair times may also be considered in order to find out whether a change in topology occurs during repair. 

In addition clusters that do not fit in size could be ruled out also by the topological similarity measure. Here, however, the practicability of this method has been demonstrated; therefore, the foci selected by the presented method have not been sorted out. The number of 400 clusters used for this analysis has been large enough that outliers, such as the mentioned foci, are not significant. On the other hand 400 clusters are manageable by interactive control of the experiment. 

The aim of this article was to demonstrate the methodological approach. In future experiments systematic studies for further parameters such as other chromatin types (e.g., euchromatin, ALU sequence regions etc.) in the environment or assignment to the follow-up proteins in the repair pathway (e.g., MRE11, Ku70, Ku80, 53BP1, Rad51, etc.) are necessary in order to understand the correlation of γH2AX clusters and other clusters formed by further recruited proteins during repair. Furthermore the application to other cell types, different repair times and radiation types (e.g., high LET ions, α-particles, β-particles etc.) would contribute to a conclusive knowledge of pathway choice and the correlation to structure and topology. This will be subject of next years’ investigations.

## 4. Materials and Methods

### 4.1. Sample Preparation

For the experiments SKBr3 cells were used, a well-established and well characterized cell line in breast cancer research [53]. It has the advantage of fast growing and usually not reaching a complete confluency (about 80% only) so that localization microscopy in the culture dishes can be performed with less background and more precision [54].

As described in details [33] SkBr3 cells were grown on coverslips until about 80% confluency. The cells were washed in 1× phosphate-buffered saline buffer (PBS) with MgCl_2_ (0.901 mM)/CaCl_2_ (0.493 mM) for 5 min and fixed in 4% formaldehyde (in 1× PBS + Mg/Ca, freshly prepared from paraformaldehyde) for 10 min at 37 °C. After washing three times in 1 PBS + Mg/Ca for 5 min, the cells were incubated in 0.2% Triton-X in 1× PBS + Mg/Ca 3 min for permeabilization followed by additional washing three times and blocking in 2% BSA in 1× PBS + Mg/Ca for 30 min.

For labeling of heterochromatin antibodies against H3K9me3 were used. H3K9me3 is traditionally associated with non-coding parts of the genome. Recent investigations [55,56] have shown that H3K9me3 is a key player in repressing lineage-inappropriate genes and shielding them from transcription. In contrast to other heterochromatin markers constitutive heterochromatin and tissue specific inactivated sites can be highlighted. 

Incubation with the primary rabbit anti-histone H3 methylation side antibody (anti histone H3 tri methyl K9—ChIP grade; Abcam plc, Cambridge, UK; concentration: 1.4 mg/L) in a humidified chamber at 37 °C for 30 min and washing three times in 1× PBS + Mg/Ca on a shaker for 5 min was then followed by incubation with the secondary goat anti-rabbit IgG H&L (Alexa Fluor^®^ 488) (Abcam plc, Cambridge, UK; concentration 4 mg/L) in a humidified chamber at 37 °C for 30 min and washing three times in 1× PBS + Mg/Ca on a shaker for 5 min. The specimen was again fixed in 2% formaldehyde at 37 °C for 10 min and washed three times in 1× PBS + Mg/Ca on a shaker for 5 min. Labeling quality was checked by experiments using the secondary antibody without specific primary antibody (Figure S4).

Incubation with the second primary mouse anti-phospho-histone H2A.X (Ser139) antibody, clone JBW301 (Merck Chemicals, Darmstadt, Germany; concentration 2 mg/L) in a humidified chamber at 4 °C for 12 h and washing three times in 1× PBS + Mg/Ca on a shaker for 5 min was then followed by incubation with the secondary goat anti-mouse antibody (Alexa Fluor^®^ 568) (Thermo Fisher Scientific, Waltham, MA, USA; concentration 4 mg/L)) in a humidified chamber at 37 °C for 30 min and washing three times in 1× PBS + Mg/Ca on a shaker for 5 min. 

Finally the chromatin was counterstained with 4′,6-DiAmidin-2-PhenylIndol (DAPI; Sigma Aldrich, now Merck, Darmstadt, Germany) for 5 min and after washing twice in 1× PBS + Mg/Ca on a shaker for 5 min embedded in 15 µL ProlongGold embedding medium (ThermoFisher Scientific, Waltham, MA, USA, ProLong Gold Antifade Mountant, P36930). After sealing the specimen can be stored at 4 °C.

### 4.2. Single Molecule Localization Microscopy (SMLM)

The microscope was built at the Light Microscopy Facility of the German Cancer Research Center in Heidelberg and is described in detail in several publications [26,27,30]. For the experiments described here two lasers (excitation at 491 nm and 561 nm) were used for the excitation of fluorescently labeled antibodies (green H3K9me3 for heterochromatin; red for γH2AX). The laser intensity was 3 KW/cm^2^ (491 nm) and 5 KW/cm^2^ (561 nm); homogeneous illumination is important for localization microscopy because blinking of dye molecules due to reversible photo-bleaching is dependent on the laser intensity. A 100× oil immersion lens with a numerical aperture of NA = 1.46 is used. The emission light captured by the objective lens is imaged onto an EmCCD-camera (Andor iXon Ultra 897, Belfast, UK). The exposure time was 100 ms per frame. Two thousand frames were captured in each channel.

### 4.3. Sample Irradiation

After culturing SkBr9 cells were irradiated by 6 MeV photons (dose rate of 3 Gy/min) at a medical linear accelerator (ARTISTE LB35) with doses of 0.1, 0.5, 1, 2, 4 and 8 Gy. SMLM images were acquired 30 min after the irradiation. For the data analysis the γH2AX foci were selected irrespective of the dose with the aim to show that the considered foci properties are independent of the dose.

## 5. Conclusions

SMLM opens new perspectives into chromatin architecture from the micro- to the nano-scale and detailed insights into molecular arrangements as repair foci. Recent investigations have shown the advantages of this method for radiation research and cell biophysics. So far microscopic analyses are usually based on images and are applied after image processing. SMLM is not necessarily dependent on image processing since the result of data acquisition is a coordinate matrix of points precisely localized. This principle difference to so far mostly established procedures allows the application of novel data evaluation procedures and mathematical concepts. In this article, the geometrical analysis of γH2AX foci towards sub-clusters was combined with persistent homology in order to classify clusters according to their heterochromatin distance. Topological characteristics of γH2AX clusters were compared independently from cell nuclei and doses applied just according to the neighborhood to heterochromatin. The detected pointillist pattern has been transferred into barcode representations of connected components and holes (Betti numbers of dimension 0 and 1) and a similarity measure has been applied leading to a similar category of clusters associated to heterochromatin and a dissimilar category of clusters not associated to heterochromatin. This proof-of-concept approach opens up new possibilities for SMLM and for a rigorous comparison of point distributions obtained for compatible objects such as repair foci and a measure of their similarity.

## Supplementary Materials

The following are available online at http://www.mdpi.com/1422-0067/19/8/2263/s1.
