# Supplementary material for: Using Persistent Homology as a New Approach for Super-Resolution Localization Microscopy Data Analysis and Classification of γH2AX Foci/Clusters
Source: Int J Mol Sci. 2018 Aug 2;19(8):2263. doi: 10.3390/ijms19082263 (PMC6121565; doi:10.3390/ijms19082263)

Supplementary Material

Figure S1: Results of SKBr3 cell nuclei not exposed to radiation treatment. The nuclei were labelled with antibodies against γH2AX (red points) but neither counter-stained with DAPI nor additionally labelled with other antibodies in order to visualize the dispersed points in the background. A) Typical SMLM image of a SKBr3 cell nucleus without cluster formation. For comparison see Figure 1 in the main text where an irradiated cell is shown with bright foci sub-divided in clusters. **B**) The relative distance frequency distribution of figure A) does not show a typical peak representative for clusters with point distances of 100-200 nm. The peak at 19 nm refers to closely neighboured points whereas the slight increase at higher distances refers to a random point distribution. **C**) Typical SMLM image of a SKBr3 cell nucleus with a few γH2AX clusters that could occur due to intrinsic repair activity of damages not induced by radiation treatment. **D**) In contrast to figure B), the relative distance frequency distribution of figure C) shows an additional peak representative for clusters with point distances of 100-200 nm.

S1 A

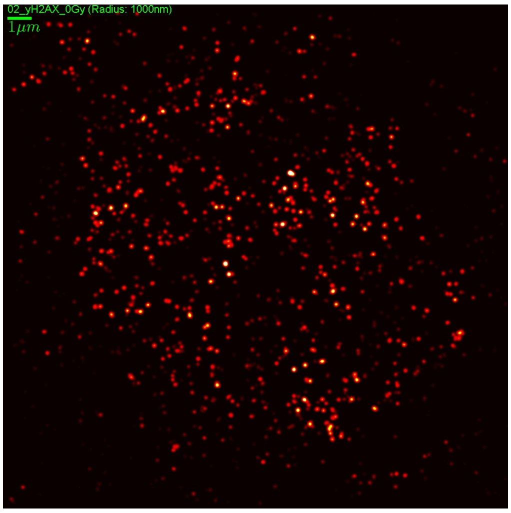

S1 B

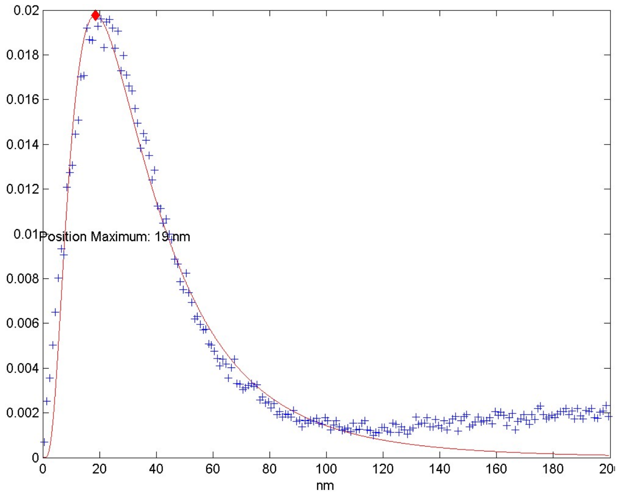

S1 C

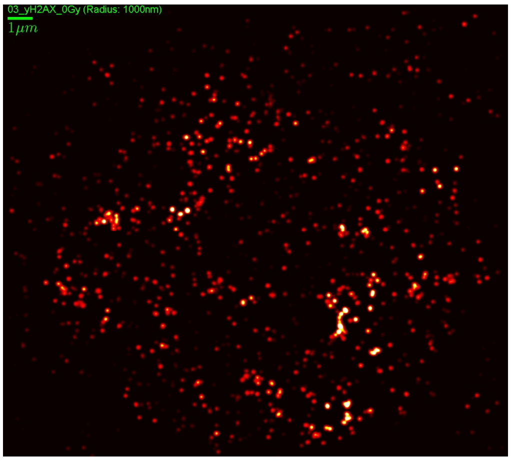

S1 D

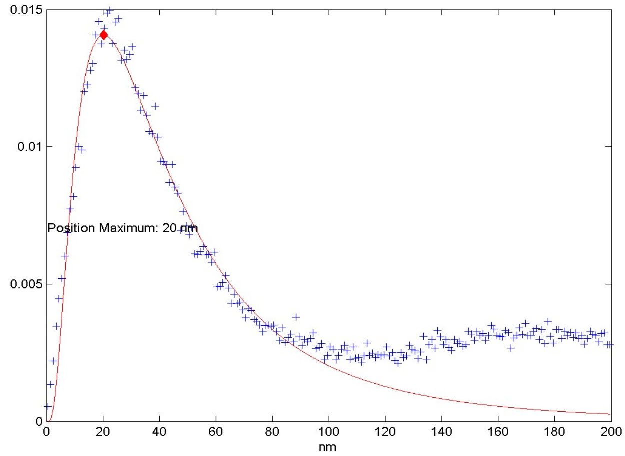

Figure S2: Boxplots of the γH2AX cluster sizes in SKBr3 cell nuclei used for topological data analysis. The data in the box show only small differences in the cluster sizes without any dose correlation.

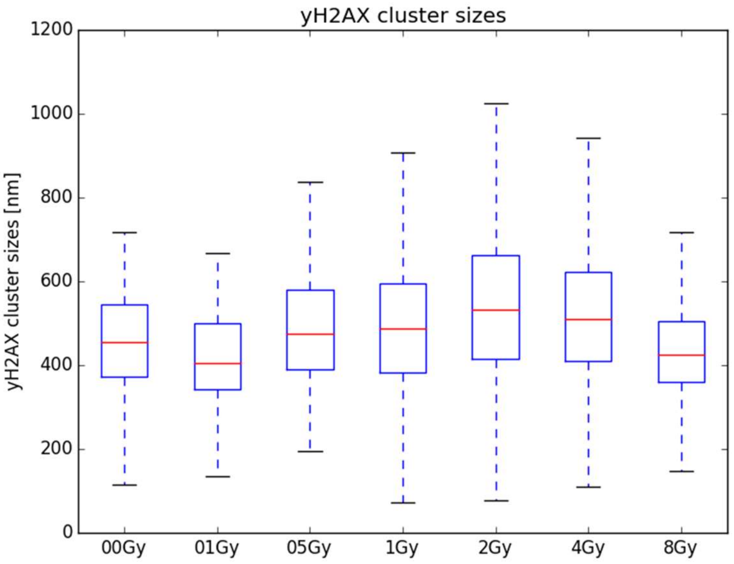

Figure S3: Example of the result of the barcode similarity measure (analog to Figure 4 in the main text). The two barcodes **A** (blue) and **B** (red) of dimension 1 (holes) have a low similarity, i.e. the overlaps of the two sets (**C, D**) of barcodes is low.

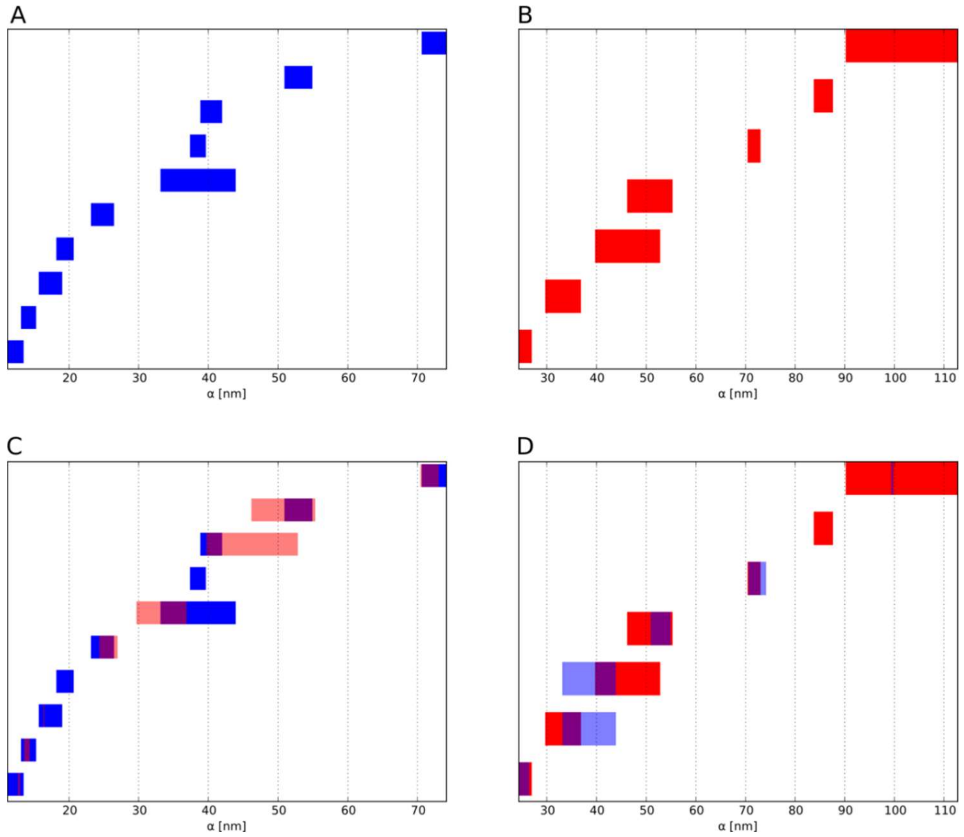

Figure S4: Examples of SMLM density images of SKBr3 cell nuclei only labeled by the secondary antibody goat anti-rabbit IgG H&L (Alexa Fluor® 488) (Abcam plc, Cambridge, UK) but neither counter-stained with DAPI nor additionally labelled with other antibodies in order to visualize the dispersed points in the background. The data reveal the quality of the staining procedure of heterochromatin requiring the specific antibodies against H3K9me3.

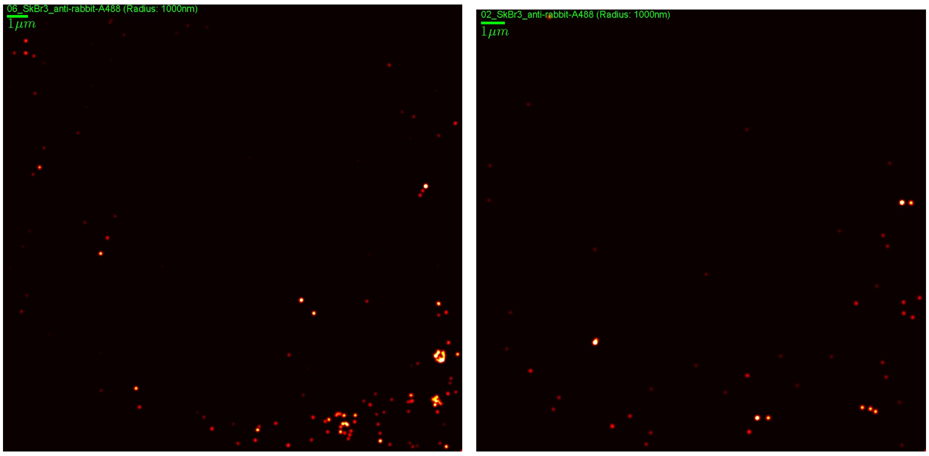

Supplement: Supplementary file 1 [file ijms-19-02263-s001.zip › ijms-318184-SI.pdf]
